# Reduced p63 expression is linked to unfavourable prognosis in muscle‐invasive urothelial carcinoma of the bladder

**DOI:** 10.1002/bco2.431

**Published:** 2024-09-10

**Authors:** Kira Furlano, Henning Plage, Sebastian Hofbauer, Sarah Weinberger, Bernhard Ralla, Annika Fendler, Florian Roßner, Simon Schallenberg, Sefer Elezkurtaj, Martina Kluth, Maximilian Lennartz, Niclas C. Blessin, Andreas H. Marx, Henrik Samtleben, Margit Fisch, Michael Rink, Marcin Slojewski, Krystian Kaczmarek, Thorsten Ecke, Stefan Koch, Nico Adamini, Sarah Minner, Ronald Simon, Guido Sauter, Joachim Weischenfeldt, Tobias Klatte, Thorsten Schlomm, David Horst, Henrik Zecha

**Affiliations:** ^1^ Department of Urology Charité – Universitätsmedizin Berlin, Corporate Member of Freie Universität Berlin, Humboldt‐Universität zu Berlin and Berlin Institute of Health Berlin Germany; ^2^ Institute of Pathology Charité – Universitätsmedizin Berlin, Corporate Member of Freie Universität Berlin, Humboldt‐Universität zu Berlin and Berlin Institute of Health Berlin Germany; ^3^ Institute of Pathology University Medical Center Hamburg‐Eppendorf Hamburg Germany; ^4^ Department of Pathology Academic Hospital Fuerth Fuerth Germany; ^5^ Department of Urology University Medical Center Hamburg‐Eppendorf Hamburg Germany; ^6^ Department of Urology Marienhospital Hamburg Hamburg Germany; ^7^ Department of Urology and Urological Oncology Pomeranian Medical University Szczecin Poland; ^8^ Department of Urology Helios Hospital Bad Saarow Bad Saarow Germany; ^9^ Department of Pathology Helios Hospital Bad Saarow Bad Saarow Germany; ^10^ Department of Urology Albertinen Hospital Hamburg Germany; ^11^ Biotech Research & Innovation Center (BRIC) University of Copenhagen Copenhagen Denmark; ^12^ Finsen Laboratory Rigshospitalet Copenhagen Denmark

**Keywords:** biomarker, immunohistochemistry, p63, tissue microarray, urothelial carcinoma

## Abstract

**Objective:**

There is a shortage of established prognostic biomarkers in bladder cancer. One candidate is tumour protein 63 (p63), a transcription factor of the p53 gene family that is expressed in the normal urothelium. Recently proposed RNA expression‐based molecular classifiers of bladder cancer identified high p63 expression as a component of a basal/squamous subtype linked to poor patient prognosis.

**Methods:**

In this study, p63 protein expression was analysed by immunohistochemistry on more than 2500 urothelial bladder carcinomas in a tissue microarray format to determine its relationship with clinicopathological parameters of disease progression and patient outcome.

**Results:**

Nuclear p63 staining was seen in all cells of normal urothelium and at elevated levels in pTaG2 tumours. The rate of p63 positive cases and the staining intensity was lower in pTaG3 tumours (93.2%, *p* < 0.0001 for pTaG3 vs. pTaG2) and markedly lower in pT2‐4 carcinomas (83.5%, *p* = 0.0120 for pT2‐4 vs. pTaG3). Within 1018 pT2‐4 carcinomas treated by cystectomy, low p63 expression was linked to nodal metastasis (*p* = 0.0028) and overall survival (*p* = 0.0005). The association with survival was independent of pT and pN (*p* = 0.0081). p63 expression was associated with GATA3 expression (*p* < 0.0001), a luminal cell type marker associated with favourable disease. A joint analysis of p63 and GATA3 did not suggest that GATA3 could provide additional prognostic information.

**Conclusion:**

The independent prognostic role of reduced p63 expression in advanced urothelial carcinomas suggests that p63 could be a useful biomarker to distinguish pT2‐4 urothelial carcinomas.

## 1 INTRODUCTION

Representing one of the most frequent malignancies worldwide, urinary bladder cancer affects a significant portion of the population. Urinary bladder cancer is the tenth most common malignant tumour type worldwide.^1^ The majority of these patients present with early‐stage, manageable cancers that are either non‐invasive or minimally invasive and have a generally positive prognosis. Nevertheless, the recurrence rate is substantial, and 20% of these patients will develop muscle‐invasive disease,^2^ necessitating further treatment strategies. For those with muscle‐invasive bladder cancer, the treatment regime often includes radiotherapy or radical cystectomy. However, outcomes are highly variable, and about 50% of these patients experience metastasis and eventually die from their disease.^3^ A better understanding of the molecular features underlying disease progression will eventually enable a better prediction of the individual patient prognosis and thus optimize treatment decisions, allowing more aggressive treatment in patients at high risk.

Tumour protein 63 (p63) is one of three transcription factors of the p53 gene family. It regulates the activity of a multitude of genes involved in growth and development of the ectoderm and ectoderm‐derived tissues, such as basal layer keratins and cell cycle control genes (summarized in Murray‐Zmijewski et al.^4^). Accordingly, p63 expression is found in basal cell layers of various epithelial tissues and in the urothelium (summarized in di Como et al.^5^, Fisher et al.^6^ and Steurer et al.^7^). p63 is often expressed in urothelial carcinomas.^5^, ^7^ In diagnostic pathology, p63 immunohistochemistry (IHC) is therefore applied to distinguish urothelial carcinomas from poorly differentiated adenocarcinomas of the prostate infiltrating the bladder.^8^ Various studies have proposed a prognostic role of p63 expression in urothelial carcinomas, but the results are highly conflicting. Several recent studies employing RNA expression screening for developing molecular classifiers of bladder cancer identified high p63 expression as a component of a basal/squamous subtype linked to poor patient prognosis.^9^, ^10^, ^11^, ^12^ Several studies using IHC for p63 detection found associations with adverse tumour features and poor patient outcome for low p63 expression by analysing 50–1086 cancers,^7^, ^13^, ^14^, ^15^, ^16^, ^17^ whereas one study on 103 cancers found an association between high p63 expression and poor clinical outcome.^18^ Six further IHC studies on 50–87 cancers could not confirm these associations.^15^, ^18^, ^19^, ^20^, ^21^, ^22^

To better understand the clinical relevance of p63 expression in urothelial carcinomas, we analysed the relationship between p63 immunostaining and clinicopathological parameters of disease progression as well as patient outcome in a cohort of more than 2700 urothelial carcinomas in a tissue microarray (TMA) format.

## 2 MATERIALS AND METHODS

### 2.1 Tissue microarrays (TMAs)

Our set of TMAs contained one sample each from 2710 urothelial tumours of the bladder archived at the Institute of Pathology, University Hospital Hamburg, Germany, Institute of Pathology, Charité Berlin, Germany, Department of Pathology, Academic Hospital Fuerth, Germany or Department of Pathology, Helios Hospital Bad Saarow, Germany, and/or treated at Department of Urology, University Hospital Hamburg, Germany, Department of Urology, Charité Berlin, Germany, Department of Urology, Helios Hospital Bad Saarow, Germany, Department of Urology, Albertinen Hospital, Hamburg, Germany and Department of Urology and Urological Oncology, Pomeranian Medical University, Szczecin, Poland between 2003 and 2021. Patients at each centre were treated according to the guidelines at the time. In brief, patients with pTa/pT1 disease underwent a transurethral resection of the bladder tumour with or without postoperative or adjuvant instillation therapy, while patients with pT2‐pT4 disease were treated by radical cystectomy. Available histopathological data, including grade, tumour stage (pT), lymph node status (pN), and status of venous (V) and lymphatic (L) invasion, are shown in Table 1. Clinical follow‐up data (overall survival [OS]: time between cystectomy and death) were available from 636 patients with pT2‐4 carcinomas treated by cystectomy (median: 15 months; range: 1–176 months). Data on GATA3 immunostaining were available from a previous study.^23^ A normal tissue TMA containing eight samples from eight different donors from each of 76 different normal tissue types was used for antibody validation. The tissues were fixed in 4% buffered formalin and then embedded in paraffin. The TMA manufacturing process has previously been described in detail.^24^, ^25^ In brief, one tissue spot (diameter: 0.6 mm) per patient was used. The use of archived remnants of diagnostic tissues for TMA manufacturing, their analysis for research purposes and patient data were according to local laws (HmbKHG, §12), and the analysis had been approved by the local ethics committee (Ethics commission Hamburg, WF‐049/09). All work has been carried out in compliance with the Helsinki Declaration.

**TABLE 1:** Patient cohort.

	Study cohort on TMA (*n* = 2710)
Follow up	636
Months	
Mean	26.7
Median	15
Tumour stage	
pTa	887 (39.2%)
pT2	462 (20.4%)
pT3	615 (27.2%)
pT4	298 (13.2%)
Tumour grade	
G2	820 (30.6%)
G3	1858 (69.4%)
Lymphnode metastasis	
pN0	734 (62.0%)
pN+	449 (38.0%)
Resection margin	
R0	595 (80.6%)
R1	143 (19.4%)
Lymphatic invasion	
L0	275 (49.5%)
L1	281 (50.5%)
Venous invasion	
V0	450 (74.4%)
V1	155 (25.6%)

*Note*: Percent in the column ‘study cohort on TMA’ refers to the fraction of samples across each category. Numbers do not always add up to 2710 in the different categories because of cases with missing data.

Abbreviation: TMA, tissue microarray.

### 2.2 Immunohistochemistry

Freshly cut TMA sections were immunostained on 1 day and in one experiment. Slides were deparaffinized with xylol, rehydrated through a graded alcohol series and exposed to heat‐induced antigen retrieval for 5 min in an autoclave at 121°C in pH 7.8 Tris‐EDTA‐Citrat (TEC) puffer. Endogenous peroxidase activity was blocked with Dako REAL Peroxidase‐Blocking Solution (Agilent Technologies, Santa Clara, CA, USA; #S2023) for 10 min. A primary antibody specific for p63 (recombinant rabbit monoclonal, MSVA‐063R, #4235‐063R, MS Validated Antibodies, Hamburg, Germany) was applied at 37°C for 60 min at a dilution of 1:150. Bound antibody was then visualized using the Dako REAL EnVision Detection System Peroxidase/DAB+, Rabbit/Mouse kit (Agilent Technologies, Santa Clara, CA, USA; #K5007) according to the manufacturer's directions. The sections were counterstained with hemalaun. The normal tissue TMA (Table S1) was also stained with an additional primary antibody (mouse monoclonal, DAK‐p63, RTU, pH 9.0, #GA662, Agilent Technologies, Santa Clara, CA, USA) in the Autostainer Link 48 (Agilent Technologies, Santa Clara, CA, USA) according to the manufacturer's directions. For the tumour TMA, the percentage of positive neoplastic cells was estimated for each tissue spot, and the staining intensity was recorded as 0, 1+, 2+ and 3+. For statistical analyses, the tumour staining results were categorized into four groups as previously described.^7^ Tumours without any staining were considered negative. Tumours with 1+ staining intensity in ≤70% of tumour cells or 2+ intensity in ≤30% of tumour cells were considered weakly positive. Tumours with 1+ staining intensity in >70% of tumour cells, 2+ intensity in 31%–70% or 3+ intensity in ≤30% were considered moderately positive. Tumours with 2+ intensity in >70% or 3+ intensity in >30% of tumour cells were considered strongly positive.

### 2.3 Statistics

Statistical calculations were performed with JMP17® software (SAS®, Cary, NC, USA). Contingency tables and the chi^2^‐test were performed to search for associations between p63 immunostaining, other molecular parameters and tumour phenotype. Survival curves were calculated according to Kaplan–Meier. The log‐rank test was applied to detect significant differences between groups. A *p*‐value of ≤0.05 was considered as statistically significant.

## 3 RESULTS

### 3.1 Technical issues

Of our 2710 urothelial carcinomas, 2461 (90.8%) were interpretable for p63. Non‐interpretable tumours were caused by a lack of unequivocal tumour cells on the TMA spots or the absence of entire tissue spots on the TMA.

### 3.2 p63 antibody validation

Among 76 different normal tissue types, p63 immunostaining was seen in squamous epithelium and urothelium, as well as in specific cell types of organs for which p63 RNA expression has also been described (https://www.proteinatlas.org/ENSG00000073282-TP63/summary/rna ^26^, ^27^, ^28^). True p63 expression in these cell types (myoepithelial cells in the salivary and breast glands, basal cells in the prostate, seminal vesicles, epididymis and respiratory epithelium, epithelial cells in the thymus, chorion and cytotrophoblast cells in the placenta) was confirmed by identical staining obtained by DAK‐p63 (Figure S1).

### 3.3 p63 in urothelial carcinomas

**FIGURE 1:**
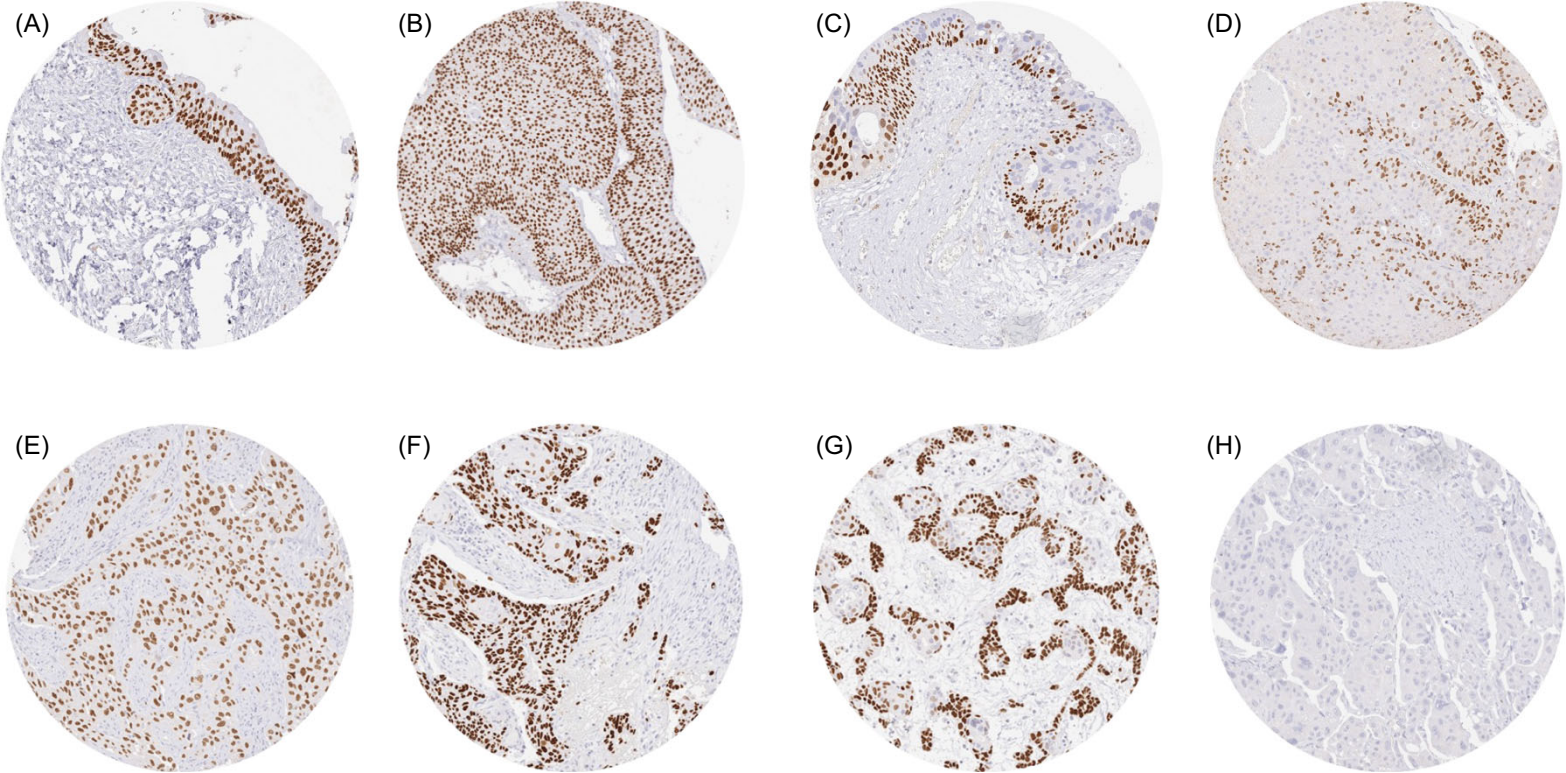
Protein 63 (p63) immunostaining in urothelial carcinomas. The panels show strong nuclear p63 staining in all cells except umbrella cells of normal urothelium (A), strong p63 staining in all cells of a low grade pTaG2 tumour (B), loss of p63 staining in most atypical cells of a urothelial carcinoma in situ (C), and loss of p63 staining in many non‐basally located tumour cells of a pTaG3 tumour (D). Samples from muscle‐invasive urothelial carcinomas show strong nuclear staining of all tumour cells (E, F), heterogeneous p63 staining predominating in basal cell layers at the invasive margin (G) and complete absence of p63 staining (H).

**FIGURE 2:**
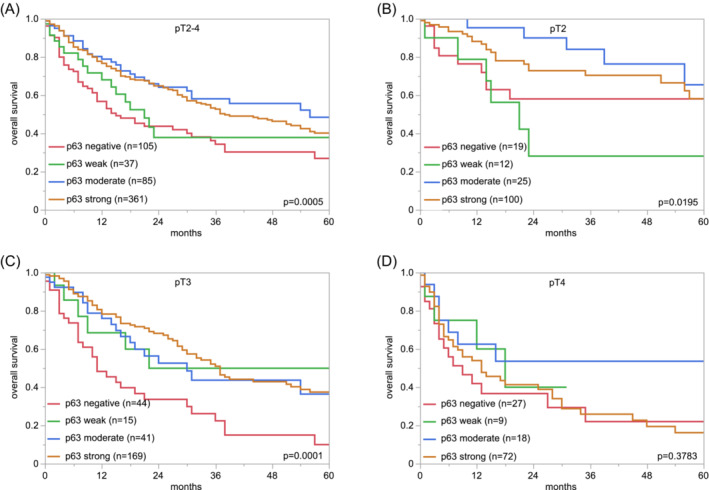
Protein 63 (p63) immunostaining and prognosis in muscle‐invasive urothelial carcinomas. (A) pT2‐4 carcinomas, (B) only pT2 carcinomas, (C) only pT3 carcinomas and (D) only pT4 carcinomas.

A strong nuclear p63 staining was regularly seen in all cells of normal urothelium. A nuclear p63 positivity—usually strong—was also observed in all pTaG2 (low and high) tumours. The rate of p63 positive cases and the staining intensity were lower, however, in pTaG3 tumours (93.2% positive, *p* < 0.0001 for pTaG3 vs. pTaG2). The p63 positivity rate further decreased in pT2‐4 carcinomas (83.5% positive, *p* = 0.0120 for pT2‐4 vs. pTaG3). Representative images are shown in Figure 1. Within the cohort of pT2‐4 carcinomas, low p63 expression was statistically associated with nodal metastasis (*p* = 0.0028) but was unrelated to pT stage and other features (Table 2). In 588 pT2‐4 carcinomas that were treated by cystectomy, low p63 staining was associated with reduced OS (*p* = 0.0005; Figure 2A). Subset analyses revealed that this association was retained in the subgroups of pT2 (*p* = 0.0195; Figure 2B) and pT3 carcinomas (*p* = 0.0001; Figure 2C) but not in the group of 126 pT4 cancers (Figure 2D). A multivariate analysis including 543 pT2‐4 carcinomas with pT and pN data revealed that p63 immunostaining predicted patient prognosis independently of pT stage and pN status (*p* = 0.0081; Table 3).

**TABLE 2:** p63 immunostaining and cancer phenotype.

		p63 immunostaining				
	*n*	Negative (%)	Weak (%)	Moderate (%)	Strong (%)	*p*‐value
All cancers	2461	11.6	5.3	9.1	74.0	
pTa G2 low	438	0.0	0.0	1.4	98.6	<0.0001
pTa G2 high	213	0.0	0.5	1.4	98.1	
pTa G3	133	6.8	6.8	14.3	72.2	
pT2	415	15.4	8.0	13.0	63.6	0.6641
pT3	554	17.5	6.1	13.7	62.6	
pT4	273	17.2	9.5	13.6	59.7	
G2	95	8.4	5.3	14.7	71.6	0.0719^a^
G3	1121	17.3	7.4	13.5	61.8	
pN0	609	14.9	6.4	14.1	64.5	0.0028^a^
pN+	409	20.3	11.0	13.9	54.8	
R0	500	15.6	9.4	13.2	61.8	0.5111^a^
R1	124	17.7	8.1	8.9	65.3	
L0	222	14.4	9.0	14.0	62.6	0.5040^a^
L1	248	18.1	11.3	11.7	58.9	
V0	376	17.6	10.6	12.0	59.8	0.2164^a^
V1	136	13.2	7.4	9.6	69.9	

Abbreviations: G, grade; L, lymphatic invasion; p63, protein 63; pN, pathological lymph node status; pT, pathological tumour stage; R, resection margin status; V, venous invasion.

^a^ Only in pT2‐4 urothelial carcinoma.

**TABLE 3:** Multivariate analysis.

Parameter		RR	95% CI	*p*‐value
pT stage	pT4 versus pT2	2.3	1.6–3.5	<0.0001
	pT3 versus pT2	1.7	1.2–2.3	
Nodal stage	pN+ versus pN0	1.8	1.3–2.3	<0.0001
p63	Negative versus strong	1.6	1.2–2.2	0.0081
	Weak versus strong	1.3	0.8–2.1	
	Moderate versus strong	0.8	0.6–1.2	

Abbreviations: CI, confidence interval; RR, risk ratio.

### 3.4 Comparison with GATA3 immunostaining

**FIGURE 3:**
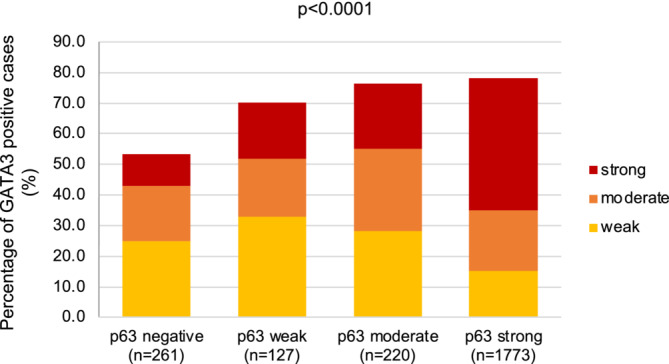
Protein 63 (p63) versus GATA3 immunostaining.

**FIGURE 4:**
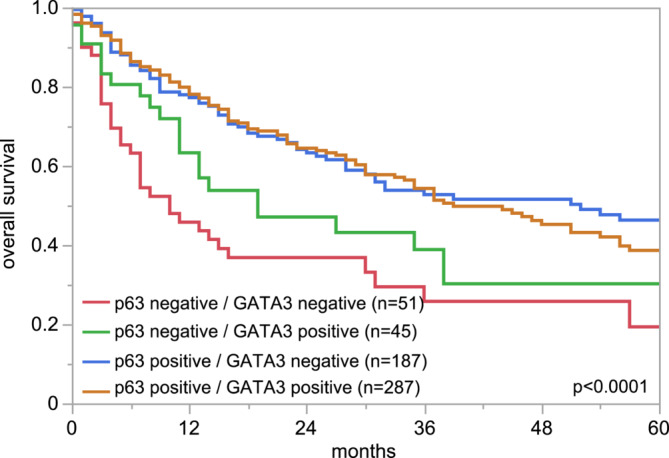
Combined protein 63 (p63) and GATA3 immunostaining and prognosis.

The relationship between p63 and GATA3 immunostaining is shown in Figure 3 for these 2381 tumours, for which data were available for both proteins. There was a strong statistical association between the expression of these two markers (*p* < 0.0001) although p63 (89.0%) was more commonly positive than GATA3 (74.9%). However, GATA3 positivity was seen in 53.3% of 261 p63 negative cancers, and p63 positivity was seen in 79.6% of 597 GATA3 negative cancers. A joint analysis of the prognostic role of p63 and GATA3 suggested that the prognostic role of p63 was independent of GATA3 (Figure 4). Especially p63 positive tumours behaved independently of their GATA3 status, and the same tendency was also seen for the smaller group of p63 negative cancers.

## 4 DISCUSSION

The results of this study demonstrate that p63 is downregulated in a fraction of urothelial neoplasms and that p63 downregulation goes along with grade and stage progression as well as unfavourable patient prognosis in these patients.

A total of 88.4% of our 2641 urothelial carcinomas showed detectable p63 expression. This is in the middle range of 21 previous studies describing p63 positivity in 41% to 100% of cases in studies on 16 to 1086 patients.^7^, ^21^, ^22^, ^29^ It appears likely that the rather significant band width of reported p63 positivity rates in the literature has been created by the use of different antibodies, staining protocols and criteria for defining p63 positivity in the respective studies. We consider our assay as suitable for p63 assessment as the assay was validated according to the guidelines of the international working group for antibody validation (IWGAV)^30^ by comparison with a second independent antibody and with RNA expression data obtained from three different publicly accessible databases in 76 different normal tissue categories (https://www.proteinatlas.org/ENSG00000073282-TP63/summary/rna ^26^, ^27^, ^28^).

The decreasing fraction of p63 positive and strongly positive cases with increasing grade of malignancy in pTa tumours are in agreement with data from our earlier study finding 100% p63 positivity in pTa G2 low and high cancers and 90% p63 positivity in pTa G3 tumours.^7^ Only a few studies have analysed p63 immunostaining in pTa urothelial carcinomas. These studies also found a comparatively high rate of p63 positive cases of 80%–100% in 62–100 carcinomas.^17^, ^21^, ^22^ Considering that p63 is highly expressed in non‐neoplastic normal urothelial cells the data from us and others are consistent with a down‐regulation of p63 during tumour progression in a fraction of urothelial carcinomas. Two major p63 isoforms—TAp63 and ΔNp63—with different cellular functions exist. While TAp63 has tumour suppressive properties, ΔNp63 owns oncogenic features (summarized in Flores^31^). In line with our findings, Papadimitriou et al. have previously identified ΔNp63 as the main p63 isoform in urothelial carcinomas and demonstrated that loss of ΔNp63 is significantly associated with a higher risk for recurrence and progression of non‐muscle‐invasive urothelial carcinomas.^32^

As compared with non‐muscle‐invasive pTa tumours, a markedly lower fraction of p63 positive cases (83.5%) was found in muscle‐invasive urothelial carcinomas. This figure is in line with the majority of earlier data. Earlier studies analysing pT2‐4 urothelial carcinomas have found p63 positivity in 32% of 98,^17^ 33% of 100,^21^ 77% of 158,^14^ and 80% of 50,^20^ 81% of 1.086,^7^ 100% of 62,^22^ and 100% of 21^33^ cancers. Only three studies have previously analysed both non‐muscle‐invasive and muscle‐invasive urothelial cancers and also found lower p63 positivity rates in muscle‐invasive cancers (32%–81%) than in non‐muscle‐invasive carcinomas (80%–96%).^7^, ^17^, ^21^ The lower rate of p63 positivity in muscle‐invasive urothelial carcinomas as compared with non‐muscle‐invasive urothelial neoplasms can be explained by a tumour‐promoting effect of reduced p63 expression. It has been shown that ΔNp63 controls epithelial–mesenchymal transition (EMT) in urothelial cancer cells through the regulation of cell adhesion, migration and motility genes.^34^ Based on this tumour suppressive and anti‐metastatic function, ΔNp63 has a potentially protective effect on tumour progression. In line with this observation, the loss of ΔNp63 was accompanied by a shift towards mesenchymal morphology and an increase in motility in squamous cell lines.^35^ However, a progressive loss of p63 during stage progression could also reflect cellular dedifferentiation. It is well known that most proteins that are downregulated during tumour development and progression do not functionally promote tumorigenicity. There is a general tendency of neoplastic cells to cease expression of non‐essential proteins during cellular dedifferentiation.^36^

The significant association of reduced p63 immunostaining with poor patient outcome, which was independent of pT and pN stage, in a cohort of 543 patients who underwent cystectomy represents the most significant finding of our study. Only a few studies have evaluated the prognostic relevance of p63 immunostaining in urothelial bladder carcinomas and have reported variable results. Three studies associated low p63 expression with poorer patient outcomes,^7^, ^15^, ^16^ one study associated high p63 expression with worse patient prognosis^18^ and one study could not find an association between p63 immunostaining and patient outcome.^20^ In addition, some studies showed a relationship between low p63 expression and adverse tumour features (i.e., pT, pN and grade),^7^, ^13^, ^14^, ^16^, ^17^ while others could not confirm these results.^13^, ^15^, ^18^, ^19^, ^21^, ^22^ However, studies interrogating the clinical utility of RNA expression‐based molecular classifiers in urothelial neoplasms identified high p63 expression as a feature of the basal/squamous subtype, which was linked to poor patient outcomes in a group of 73 to 937 urothelial bladder cancer patients.^9^, ^10^, ^11^, ^12^ A phenomenon that may partially explain the perceived discrepancy between our data and these RNA studies may be the ‘RNA subtype’ of ‘stroma‐rich’ urothelial neoplasms, which is comprised of cases with low p63 expression and the worst clinical outcome.^37^ Moreover, high expression of TAp63 was associated with favourable patient outcomes in one RNA‐based study on 36 patients with urothelial carcinomas.^38^

A high level of GATA3 expression had earlier been suggested as a hallmark of the luminal subtype, which was linked to favourable disease outcomes in RNA‐based bladder cancer classifiers.^10^, ^11^, ^12^, ^37^ That a joint analysis of p63 and GATA3 did not suggest a relevant contribution of GATA3 to the prognostic information further suggests that RNA based classifiers cannot easily be converted into a small set of IHC markers that will provide clinically relevant information on bladder cancer prognosis. It is noteworthy, that assessment of tumour aggressiveness may be more difficult in muscle‐invasive urothelial carcinomas than in many other tumours. To date, neither a single molecular feature nor a molecular panel has been established to assess bladder cancer aggressiveness in routine practice. Because not even the histologic grade has a documented prognostic role, it is recommended to avoid histologic grading in pT2‐4 urothelial cancers.^39^ Nuclear accumulation of p53 protein, a strong prognostic feature in many cancers, has failed to be linked to poor outcomes in our patient cohort^40^ and in two meta‐analyses summarizing data from more than 10 000 urothelial cancer patients.^41^, ^42^

In summary, the independent prognostic role of reduced p63 expression in advanced urothelial carcinomas is of interest given the shortage of established prognostic features in this tumour entity. It appears possible that p63 analysis, most likely in combination with other parameters, could emerge as a clinically useful tool in the evaluation of pT2‐4 urothelial carcinomas.

## AUTHOR CONTRIBUTIONS

**Kira Furlano, Henrik Zecha, Henning Plage, Martina Kluth, Ronald Simon, Thorsten Schlomm and Guido Sauter:** Contributed to conception, design, data collection, data analysis and manuscript writing. **Kira Furlano, Henning Plage, Maximilian Lennartz, Niclas C. Blessin, Sarah Minner, Florian Roßner, Simon Schallenberg, Sefer Elezkurtaj, Andreas H. Marx, Henrik Samtleben, Stefan Koch, and David Horst:** Participated in pathology data analysis and data interpretation. **Sebastian Hofbauer, Sarah Weinberger, Bernhard Ralla, Annika Fendler, Florian Roßner, Simon Schallenberg, Sefer Elezkurtaj, Andreas H. Marx, Henrik Samtleben, Margit Fisch, Michael Rink, Marcin Slojewski, Krystian Kaczmarek, Thorsten Ecke, Stefan Koch, Nico Adamini, Tobias Klatte and David Horst:** Collection of samples. **Ronald Simon and Martina Kluth:** Data analysis. **Henrik Zecha, Kira Furlano, Henning Plage, Thorsten Schlomm, Ronald Simon and Guido Sauter:** Study supervision. All authors agree to be accountable for the content of the work.

## CONFLICT OF INTEREST STATEMENT

The recombinant rabbit monoclonal p63 antibody, clone MSVA‐063R, was obtained from MS Validated Antibodies GmbH, Hamburg, Germany (owned by a family member of GS).

## Supporting information


**Table S1.** Normal tissue microarray.

**Figure S1.** **Immunohistochemical validation by comparison of antibodies.** The panels show a concordance of immunostaining results obtained by two independent p63 antibodies (MSVA‐063R, DAK‐63). Using MSVA‐063R, a nuclear positivity was seen at variable intensity in epithelial cells of the uterine cervix (A) and the urothelium (B), basal cells of respiratory epithelium (C), prostate (D), seminal vesicle (E), and the epididymis (F), myoepithelial cells of the breast (G) and of salivary glands (H), cytotrophoblast cells (I) and chorion cells (K) of the placenta, epithelial cells of the thymus (L), and a small fraction of lymphocytes (M). Using clone DAK‐63, p63 staining was seen in identical cell types of uterine cervix (N), urothelium (O), respiratory epithelium (P), prostate (Q), seminal vesicle (R), epididymis (S), breast (T), salivary glands (U), placenta (V), chorion (W), thymus (X), and lymphocytes (Y). The images A‐M and N‐Y are from consecutive tissue sections.

## Data Availability

All data generated or analysed during this study are included in this published article.
