# Supplementary figures and images for: Reduced p63 expression is linked to unfavourable prognosis in muscle‐invasive urothelial carcinoma of the bladder
Source: BJUI Compass. 2024 Sep 10;5(11):1081–9. doi: 10.1002/bco2.431 (PMC11557268; doi:10.1002/bco2.431)

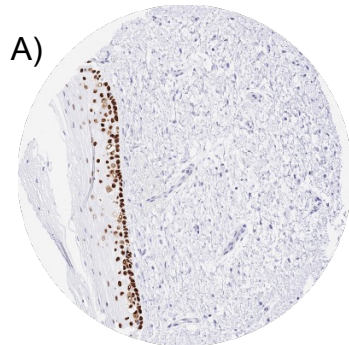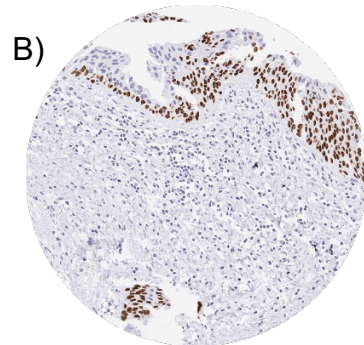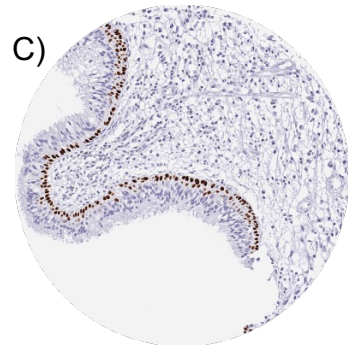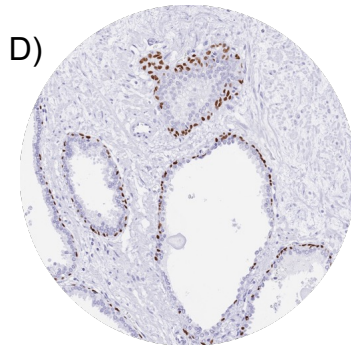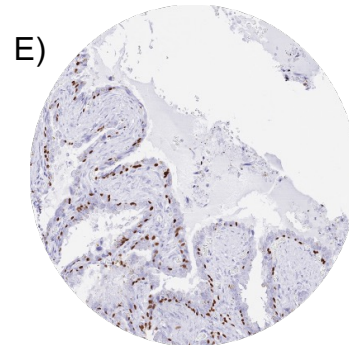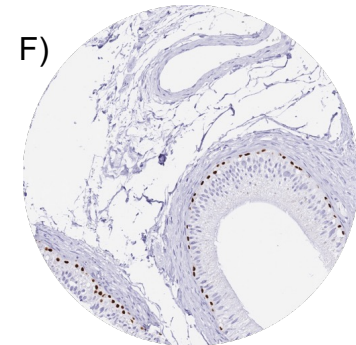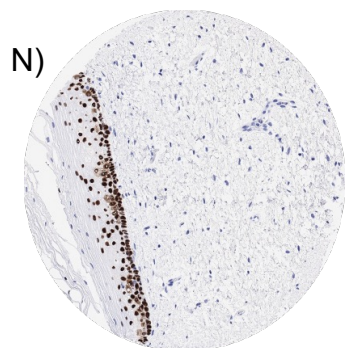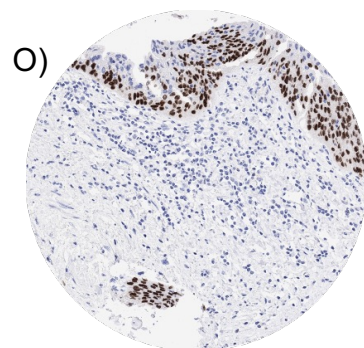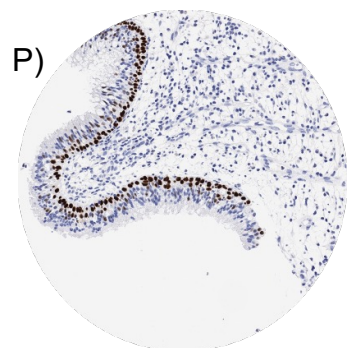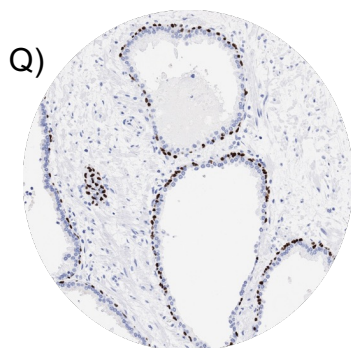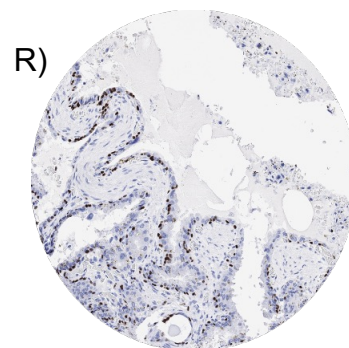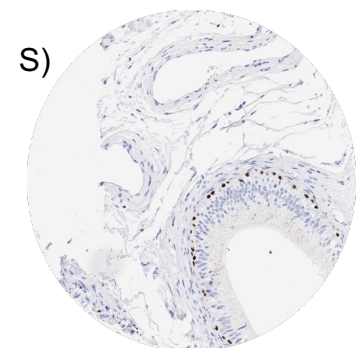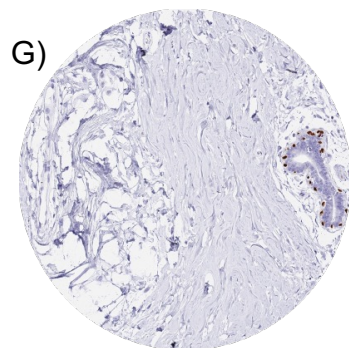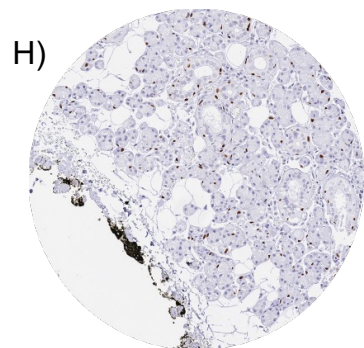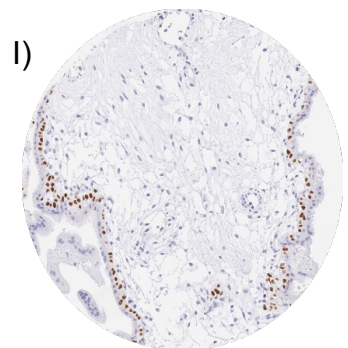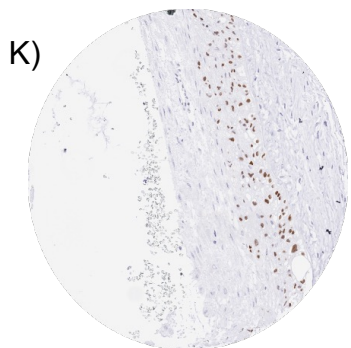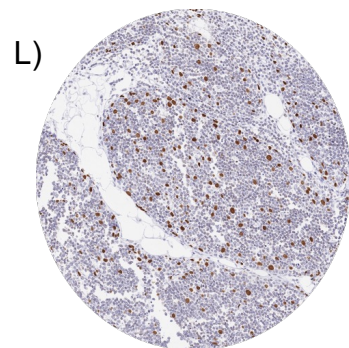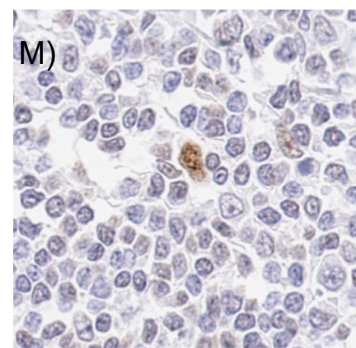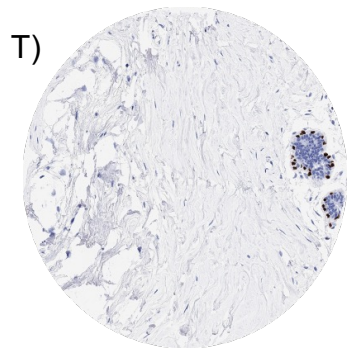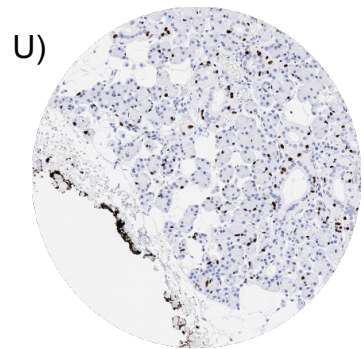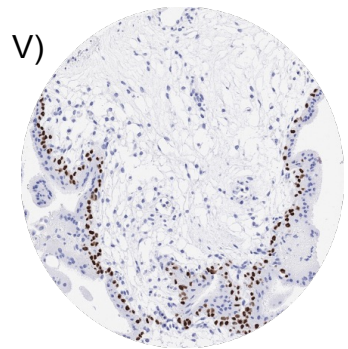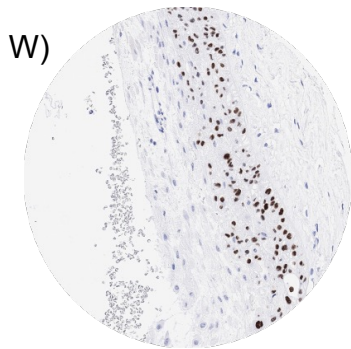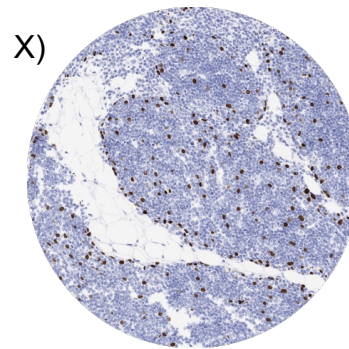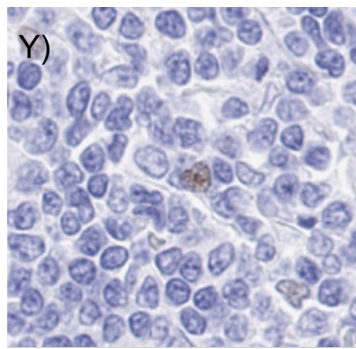

Supplement: Supplementary file 2 — Figure S1. Immunohistochemical validation by comparison of antibodies. The panels show a concordance of immunostaining results obtained by two independent p63 antibodies (MSVA‐063R, DAK‐63). Using MSVA‐063R, a nuclear positivity was seen at variable intensity in epithelial cells of the uterine cervix (A) and the urothelium (B), basal cells of respiratory epithelium (C), prostate (D), seminal vesicle (E), and the epididymis (F), myoepithelial cells of the breast (G) and of salivary glands (H), cytotrophoblast cells (I) and chorion cells (K) of the placenta, epithelial cells of the thymus (L), and a small fraction of lymphocytes (M). Using clone DAK‐63, p63 staining was seen in identical cell types of uterine cervix (N), urothelium (O), respiratory epithelium (P), prostate (Q), seminal vesicle (R), epididymis (S), breast (T), salivary glands (U), placenta (V), chorion (W), thymus (X), and lymphocytes (Y). The images A‐M and N‐Y are from consecutive tissue sections. [file BCO2-5-1081-s001.pdf]
